# Characterization and quantitation of fluorescent Gag virus-like particles

**DOI:** 10.1186/1753-6561-7-S6-P62

**Published:** 2013-12-04

**Authors:** Sonia Gutiérrez-Granados, Laura Cervera, Francesc Gòdia, María Mercedes Segura

**Affiliations:** 1 Departament d'Enginyeria Química, Universitat Autònoma de Barcelona, Bellaterra, Barcelona, 08193, Spain

## Background

Upon expression, the Gag polyprotein of HIV-1 spontaneously assembles giving rise to enveloped virus-like particles (VLPs). These particulate immunogens offer great promise as HIV-1 vaccines. In order to develop robust VLP manufacturing processes, the availability of simple, fast and reliable quantitation tools is crucial. Traditionally, commercial p24 ELISA kits are used to estimate Gag VLP concentrations. However, this quantitation technique is time-consuming, laborious, costly and prone to methodological variability. Reporter proteins are frequently used during process development to allow a straightforward monitoring and quantitation of labeled products. This alternative was evaluated in the present work by using a Gag-GFP fusion construct.

## Materials and methods

Generation of fluorescent VLPs was carried out by transient transfection of HEK 293 suspension cells with a plasmid coding for Gag fused to GFP (NIH AIDS Reagent Program). VLP budding from producer cells was visualized by electron microscopy (JEM-1400, Jeol) and confocal fluorescence microscopy (Fluoview^® ^FV1000, Olympus, Japan). A purified standard of Gag-GFP VLP material was obtained by ultracentrifugation through a sucrose cushion and fully characterized. SDS-PAGE, Western blot, size-exclusion chromatography (SEC), nanoparticle tracking analysis (NTA, NanoSight^®^, UK) and transmission electron microscopy (TEM) were used for VLP characterization. The standard VLP material was used for the development and validation of a Gag-GFP VLP quantitation technique based on fluorescence. Viral particle titers estimated using this method were compared with those obtained by p24 ELISA (Innotest^®^, Innogenetics, Belgium), densitometry, TEM and NTA.

## Results

**Figure 1:**
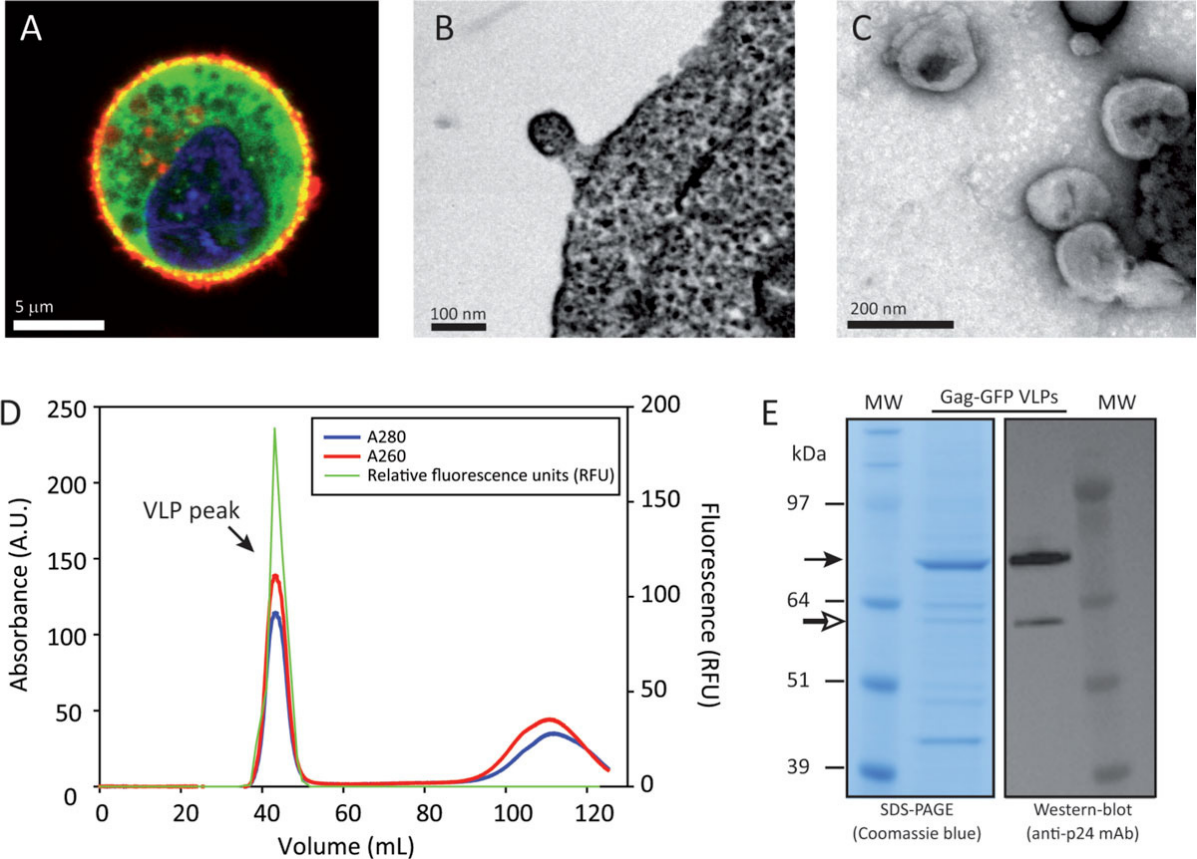
**Characterization of the purified standard Gag-GFP VLP material**. **(A) **Confocal fluorescence microscopy image of a HEK 293 producer cell expressing green fluorescent Gag-GFP molecules. The lipid membrane is stained with Cell Mask™ (red) and the cell nucleus with Hoechst (blue). **(B) **TEM image of an ultrathin section showing VLP budding from HEK 293 producer cells. **(C) **Negatively stained Gag-GFP VLPs in the purified standard material. **(D) **Size exclusion chromatogram of the standard Gag-GFP VLP material. **(E) **SDS-PAGE and Western-blot analyses of the standard VLP material. Full and empty arrows represent Gag-GFP protein and Gag-GFP fragment, respectively. Abbreviations: MW, molecular weight standard.

Upon transfection, Gag-GFP was expressed in the cytoplasm of the producer cells and accumulated in the vicinity of the plasma membrane where the budding process takes place. Upon staining with Cell Mask™, co-localization of green (Gag-GFP molecules) and red (lipid membrane) fluorescence was observed in yellow (Figure 1A). VLP budding was also visualized in TEM images of ultrathin sections of HEK 293 producer cells (Figure 1B).

A purified Gag-GFP VLP standard material was obtained by harvesting VLPs from cell culture supernatants of transfected HEK 293 cells by low speed centrifugation followed by VLP pelleting through a 30% sucrose cushion. The purity of the standard material was analyzed by SEC. The SEC chromatogram showed a single peak eluting in the column void volume (V_0 _= 44 mL) as determined by UV and fluorescence analyses of collected fractions (Figure 1D). The A260/A280 ratio was 1.24 which is consistent with reported ratios for purified retroviral particles [1]. The standard VLP material was further characterized using different techniques. Particle morphology was analyzed by TEM. Roughly spherical viral particles surrounded by a lipid envelope and containing an electro-dense core could be observed (Figure 1C). The mean VLP diameter according to TEM analysis was determined to be 141 ± 22 nm (*n *= 100), which is the expected size of Gag-GFP VLPs as they resemble immature HIV particles that are larger than wild-type HIV-1 virions [2]. NTA analyses of the standard material showed that the most frequent particle size value (statistical mode) was 149 ± 5 nm, which is consistent with our TEM results. SDS-PAGE analysis of the standard VLP material (Figure 1E) was performed. Approximately, 65% of the total protein loaded in the gel corresponded to Gag-GFP (Figure 1E, full arrow), the major HIV-1 VLP structural protein. The other minor bands should correspond to cellular proteins derived from host cells as retroviral particles are known to promiscuously incorporate a significant amount of host proteins [3,4]. A Gag-GFP band of the expected molecular weight (~81 kDa) was specifically detected using an anti-p24 mAb by Western blot analysis (Figure 1E, full arrow). The presence of a Gag-GFP fragment (Figure 1E, empty arrow), representing only 5% of the total Gag-GFP loaded, was also observed in the gel.

A fluorescence-based quantitation method for Gag-GFP VLPs was developed [5]. Validation of the quantitation assay was carried out according to International Conference Harmonization (ICH) guidelines [6]. The validation parameters evaluated included specificity, linearity, quantitation range, limit of detection, precision, and accuracy [5]. All validation parameters met the criteria for analytical method validation. Some parameters were also studied in parallel for p24 ELISA for comparison purposes (Table 1). Both techniques specifically detected Gag-GFP. Even though the p24 ELISA assay showed to be more sensitive for Gag-GFP detection, the fluorescence-based method was more precise and showed to be linear in a wider range. In addition, the developed quantitation method required less time and was considerably less expensive than the traditional p24 ELISA method used for Gag VLP quantitation. Finally, the standard VLP material was quantified using several methods. In order to compare the concentration of Gag-GFP in μg/mL as determined by the fluorescence-based method, ELISA and densitometry with the titers obtained by TEM and NTA analyses which are given in particles/mL, it was assumed that a Gag VLP contains 2500 Gag molecules as previously reported [7]. All concentration values, regardless of the quantitation technique used, were in close agreement within an expected range. These results support the reliability of the fluorescence-based method developed [5].

**Table 1:** Comparison between the fluorescence-based quantitation method and the p24 ELISA assay

	Fluorescence-based method	p24 ELISA assay
Specificity	Gag-GFP fusion protein	Gag-GFP fusion protein
Linear range	7 to 1000 RFU (10 to 3600 ng of p24/mL)	10 to 300 pg of p24/mL
Precision	~2% CV	~10% CV
Limit of detection	10 ng/mL of p24	10 pg/mL of p24
Time (96 samples)	~1.5 h	~4 h
Price (96 samples)	~10 €	~400 €

RFU: Relative fluorescence units

CV: Coefficient of variation

## Conclusions

Due to the flexibility of the retrovirus particle assembly process, fluorescently tagged Gag VLPs can be easily generated by expressing Gag as a fusion construct with GFP. Although fluorescently labeled Gag has mainly been used to study retrovirus replication in living cells, this attractive feature is exploited in our laboratory to facilitate the monitoring and quantitation of Gag VLPs. A purified standard VLP material was obtained and fully characterized. VLPs in the standard material showed to be of the expected size, morphology and with a composition consistent with immature HIV-1 particles. A fast, reliable and cost-effective quantitation method based on fluorescence was developed and validated using the standard VLP material. The fluorescence-based quantification method should facilitate the development and optimization of bioprocessing strategies for Gag-based VLPs.
